# Three new species of *Colletotrichum* (*Glomerellaceae*, *Glomerellales*) based on morphology and multilocus phylogeny from Fujian, China

**DOI:** 10.3897/mycokeys.139.208158

**Published:** 2026-08-25

**Authors:** Xi-Gang Yan, Yang Jiang, Xing-Sheng Wang, Jin-Yan Sheng, Yue Zhang, Cong-Cong Ai, Xiu-Guo Zhang, Shi Wang

**Affiliations:** 1 College of Life Sciences, Shandong Normal University, Jinan, 250358, China College of Life Sciences, Shandong Normal University Jinan China; 2 Shandong Provincial Key Laboratory for Biology of Vegetable Diseases and Insect Pests, College of Plant Protection, Shandong Agricultural University, Taian, 271018, China Shandong Provincial Key Laboratory for Biology of Vegetable Diseases and Insect Pests, College of Plant Protection, Shandong Agricultural University Taian China

**Keywords:** *
Ascomycetes
*, fungal diversity, new taxa, phylogenetic analysis, taxonomy

## Abstract

*Colletotrichum* species are well-known plant pathogens, and many also exist as endophytes or saprobes on diverse plant hosts. In this study, three novel *Colletotrichum* species were isolated from diseased leaves of *Millettia rugosa*, *Paederia scandens*, and *Pleioblastus fortunei* in Fujian Province, China, and taxonomically characterized using an integrative taxonomic approach. Species boundaries were delineated through combined molecular phylogenetic reconstruction and morphological comparison with closely allied taxa. Phylogenetic reconstructions were carried out based on concatenated sequence datasets of five loci (ITS, *gapdh*, *chs-1*, *act*, and *tub2*) using both maximum likelihood (ML) and Bayesian inference (BI) analytical methods. The results indicate that these three species form highly supported, independent monophyletic lineages clearly distinguishable from all previously described *Colletotrichum* species. Morphological assessment of colony traits, conidiogenous cells, and conidial morphology further confirms their taxonomic novelty by identifying stable phenotypic differences relative to phylogenetically close relatives. Consequently, three new species of *Colletotrichum* are formally proposed: *C. millettii*, *C. pleioblasti*, and *C. wuyishanense*. Phylogenetically, *C. wuyishanense* nests within the *C. destructivum* species complex, whereas *C. millettii* and *C. pleioblasti* represent two distinct monophyletic lineages nested within the genus without assignment to any formally described species complex. This study implies that additional undescribed *Colletotrichum* taxa remain to be uncovered from subtropical plant hosts across China, which will enrich genus-level species diversity and support native plant conservation and anthracnose disease management.

## Introduction

*Colletotrichum* (*Glomerellaceae*, *Glomerellales*, *Sordariomycetes*) represents one of the most economically important ascomycetous fungal genera worldwide. Its taxonomy has undergone extensive revisions, and the “one fungus, one name” nomenclatural principle synonymized the former sexual morph genus *Glomerella* with *Colletotrichum* (Hawksworth 2011). Species identification relying solely on morphology is frequently unreliable due to overlapping phenotypic features and intraspecific plasticity (Vieira et al. 2020; Bhunjun et al. 2021). Modern polyphasic taxonomy, combining morphological observations with multilocus phylogenetic analyses based on ITS, *gapdh*, *chs-1*, *act*, and *tub2*, has greatly advanced species delimitation and recognized 16 major species complexes plus numerous singleton species within this genus (Bhunjun et al. 2021). Among these, the *C. destructivum* species complex constitutes a well-supported monophyletic hemibiotrophic clade comprising 36 described species, many of which are host-specific pathogens of herbaceous crops, medicinal plants, and forages in temperate and subtropical regions and cause severe anthracnose diseases (Damm et al. 2014). *Colletotrichum* species exhibit diverse ecological lifestyles. While many members are well-known hemibiotrophic plant pathogens, numerous species also thrive as asymptomatic endophytes or saprobes in plant tissues, displaying flexible trophic strategies across wide host ranges (Newfeld et al. 2025).

In the present study, diseased leaf samples were collected in Fujian Province, China. *Colletotrichum* strains were isolated and subjected to morphological characterization and multilocus phylogenetic reconstruction. Three novel species are described herein. This work expands knowledge of *Colletotrichum* diversity in Fujian and contributes to the global taxonomic understanding of the genus.

## Materials and methods

### Sample collection and isolation

Sample collection for the present research was conducted in Fujian Province through multiple field surveys in 2024, with the majority of specimens obtained from leaves showing disease symptoms and rot lesions. Detailed information related to sample collection was recorded in accordance with the previously reported protocol (Rathnayaka et al. 2025), and all collected specimens were transported to the laboratory of Shandong Normal University for subsequent processing. Multiple fungal strains were found colonizing the sampled leaf tissues, so single-spore separation and tissue isolation procedures described by Wang et al. (2023) and Zhang et al. (2025) were adopted to obtain purified fungal cultures. For single-spore isolation, conidial suspensions were uniformly distributed on water agar plates and cultured at a constant temperature of 25 °C, after which germinated single conidia were individually picked with a sterile fine needle under a stereomicroscope and inoculated onto fresh potato dextrose agar (PDA) medium. For tissue isolation, 5 × 5 mm leaf fragments were cut from symptomatic and necrotic areas of diseased leaves and placed in individual sterile vessels for surface sterilization. The leaf tissues were first soaked in 75% ethanol for 1 min for preliminary disinfection and rinsed once with sterile distilled water, then treated with 5% sodium hypochlorite solution for 30 s, followed by three successive washes with sterile water to remove residual disinfectant. After air-drying on sterile filter paper, four processed leaf fragments were symmetrically placed on freshly prepared PDA medium (composed of 200 g potato, 20 g glucose, 20 g agar, and 1,000 mL distilled water, pH adjusted to 7.0), with the lesion side in full contact with the medium surface. All culture plates were subsequently sealed, marked with corresponding sample information, and incubated at 25 °C, with fungal growth observed and recorded daily. Agar blocks containing hyphae collected from the marginal areas of emerging colonies were transferred to new PDA plates for strain purification, and the cultures were maintained for 1–2 weeks to obtain stable pure fungal colonies.

### Morphological and cultural characterization

Colony morphological characteristics were photographed after 7 and 14 days of cultivation using a Canon PowerShot G7 X digital camera. Conidia were harvested from 7–14-day-old PDA cultures incubated at 25 °C in darkness. Mycelial debris was removed by filtration through sterile gauze. Conidial suspensions were washed twice with sterile distilled water by centrifugation (5,000 rpm, 3 min) and subsequently adjusted to approximately 2–3 × 10^5^ conidia/mL with sterile distilled water. A 20 μL aliquot of conidial suspension was placed onto the hydrophobic surface of glass coverslips to induce appressorium formation. The coverslips were placed in a moist chamber and incubated at 25 °C in darkness. Appressorium morphology was observed and measured after 24 h of incubation using light microscopy; measurements were taken from at least 30 mature structures when present. For the observation of fungal micromorphology, conidia were picked with sterile dissecting needles and placed on glass slides with a drop of lactic acid-phenol mounting solution (lactic acid:phenol:glycerol water = 1:1:2:1), then covered with coverslips to prepare temporary microscopic mounts. The mounts were observed under an Olympus BX53 light microscope fitted with an Olympus DP80 high-definition color digital camera to examine key microstructural features, including conidiomata, conidiophores, conidiogenous cells, conidia, and their appendages. Digimizer software (https://www.digimizer.com/) was used to measure the aforementioned microstructures, with a minimum of 25 replicate measurements taken for each morphological trait. All fungal strains were preserved in 10% sterilized glycerol at 4 °C for subsequent experimental investigations. Voucher specimens from this study were deposited in the Herbarium Mycologicum Academiae Sinicae (HMAS), while viable fungal cultures were deposited in the Shandong Agricultural University Culture Collection (SAUCC). The morphological descriptions and taxonomic information of the new species have been uploaded to Fungal Names (https://nmdc.cn/fungalnames/, accessed 14 July 2026).

### DNA extraction, PCR amplification, and sequencing

Genomic DNA was extracted from growing colonies on PDA medium using the CTAB method (Guo et al. 2000). Polymerase chain reaction (PCR) was performed to amplify five target gene regions, namely ITS, *act*, *gapdh*, *chs-1*, and *tub2*. The specific primers and PCR cycling programs employed are detailed in Table 1. All PCR amplifications were carried out in a total reaction volume of 25 μL, consisting of 12.5 μL of 2× Hieff Canace® Plus PCR Master Mix (with dye, Yeasen Biotechnology, Shanghai, China; Cat. No. 10154ES03), 1 μL of forward primer, 1 μL of reverse primer, 2 μL of genomic DNA template, and nuclease-free distilled water to bring the volume to 25 μL. PCR amplicons were separated by 2% agarose gel electrophoresis, and amplification success was verified via ultraviolet (UV) transillumination for band visualization. Target DNA fragments were then recovered from the gels using a Gel Extraction Kit (Shandong Sparkjade Biotechnology Co., Ltd.; Cat. No. AE0101-C). Purified PCR products were sent to Sangon Biotech Co., Ltd. (Shanghai, China) for Sanger sequencing. Raw sequencing data were subsequently analyzed and assembled into contigs using MEGA v.7.0 (Kumar et al. 2016). The most recent taxonomic information pertaining to the genus *Colletotrichum* was retrieved from Index Fungorum and combined with the taxonomic information of the three novel species described in the present study to compile the species dataset used for subsequent analyses.

**Table 1.** Gene loci and corresponding PCR primers and programs used in this study.

**Locus**	**PCR primers**	**Sequence (5’ – 3’)**	**PCR cycles**	**References**
ITS	ITS5	GGAAGTAAAAGTCGTAACAAGG	(95 °C: 30 s, 55 °C: 30 s, 72 °C: 45 s) × 29 cycles	White et al. 1990
	ITS4	TCCTCCGCTTATTGATATGC		
*gapdh*	GDF	GCCGTCAACGACCCCTTCATTGA	(95 °C: 30 s, 61.9 °C: 30 s, 72 °C: 1 min) × 35 cycles	Guerber et al. 2003
	GDR	GGGTGGAGTCGTACTTGAGCATGT		
*act*	ACT-512F	ATGTGCAAGGCCGGTTTCGC	(95 °C: 30 s, 60.3 °C: 30 s, 72 °C: 1 min) × 35 cycles	Carbone and Kohn 1999
	ACT-783R	TACGAGTCCTTCTGGCCCAT		
*chs-1*	CHS-79F	TGGGGCAAGGATGCTTGGAAGAAG	(95 °C: 30 s, 59.4 °C: 30 s, 72 °C: 1 min) × 35 cycles	Carbone and Kohn 1999
	CHS-345R	TGGAAGAACCATCTGTGAGAGTTG		
*tub2*	Bt2a	GGTAACCAAATCGGTGCTGCTTTC	(95 °C: 30 s, 61.3 °C: 30 s, 72 °C: 1 min) × 35 cycles	Glass and Donaldson 1995
	Bt2b	ACCCTCAGTGTAGTGACCCTTGGC		

### Phylogenetic analyses

In the present study, newly generated sequences were deposited in the NCBI GenBank database. Relevant reference sequences were retrieved from the National Center for Biotechnology Information (NCBI) in accordance with the latest taxonomic publications focusing on the genus *Colletotrichum*. All newly generated sequences, together with reference sequences, were aligned using the MAFFT 7 online platform (Katoh et al. 2019) and manually adjusted. Multilocus phylogenetic analyses were performed using two statistical methods: Bayesian inference (BI) and maximum likelihood (ML). First, MrModeltest v.2.3 was used to select the optimal nucleotide substitution model for each gene partition based on the Akaike information criterion (AIC), providing a foundation for subsequent BI phylogenetic analysis. Subsequently, both ML and BI analyses were conducted on the CIPRES Science Gateway portal (https://www.phylo.org/, accessed 22 December 2025) or using corresponding offline analytical software (Miller et al. 2012). ML analysis was carried out using the RAxML-HPC2 tool on XSEDE (version 8.2.12), with 1,000 rapid bootstrap replicates performed under the GTRGAMMA evolutionary model and other default parameter settings (Stamatakis 2014). BI analysis was conducted via XSEDE (version 3.2.7a) using the Markov chain Monte Carlo (MCMC) algorithm with an automatic termination rule; phylogenetic trees were generated once the average standard deviation of split frequencies dropped below 0.01 (Ronquist and Huelsenbeck 2003; Ronquist et al. 2012). Finally, all inferred phylogenetic trees were visualized using FigTree v.1.4.4 (http://tree.bio.ed.ac.uk/software/figtree, accessed 28 April 2026) and Interactive Tree of Life (iTOL, https://itol.embl.de/, accessed 30 April 2026), followed by layout optimization and diagram refinement in Adobe Illustrator CC 2023 (Letunic and Bork 2021). The names of isolates obtained in this study were labeled in red on all phylogenetic topologies. The species information used in this research is presented in Suppl. material 1.

The Genealogical Concordance Phylogenetic Species Recognition (GCPSR) model, with a pairwise homoplasy index (PHI) test, was used to analyze the new taxa and their phylogenetically closest neighbors (Quaedvlieg et al. 2014). The PHI test was performed in SplitsTree v.4.14.6 (Huson 1998; Huson and Bryant 2006) with a concatenated dataset to determine the level of recombination among phylogenetically closely related species. A pairwise homoplasy index below the 0.05 threshold (*Φw* < 0.05) indicated the presence of significant recombination in the dataset. The relationships among closely related species were visualized by constructing a split network.

## Results

### Phylogenetic analyses

**Figure 1.**
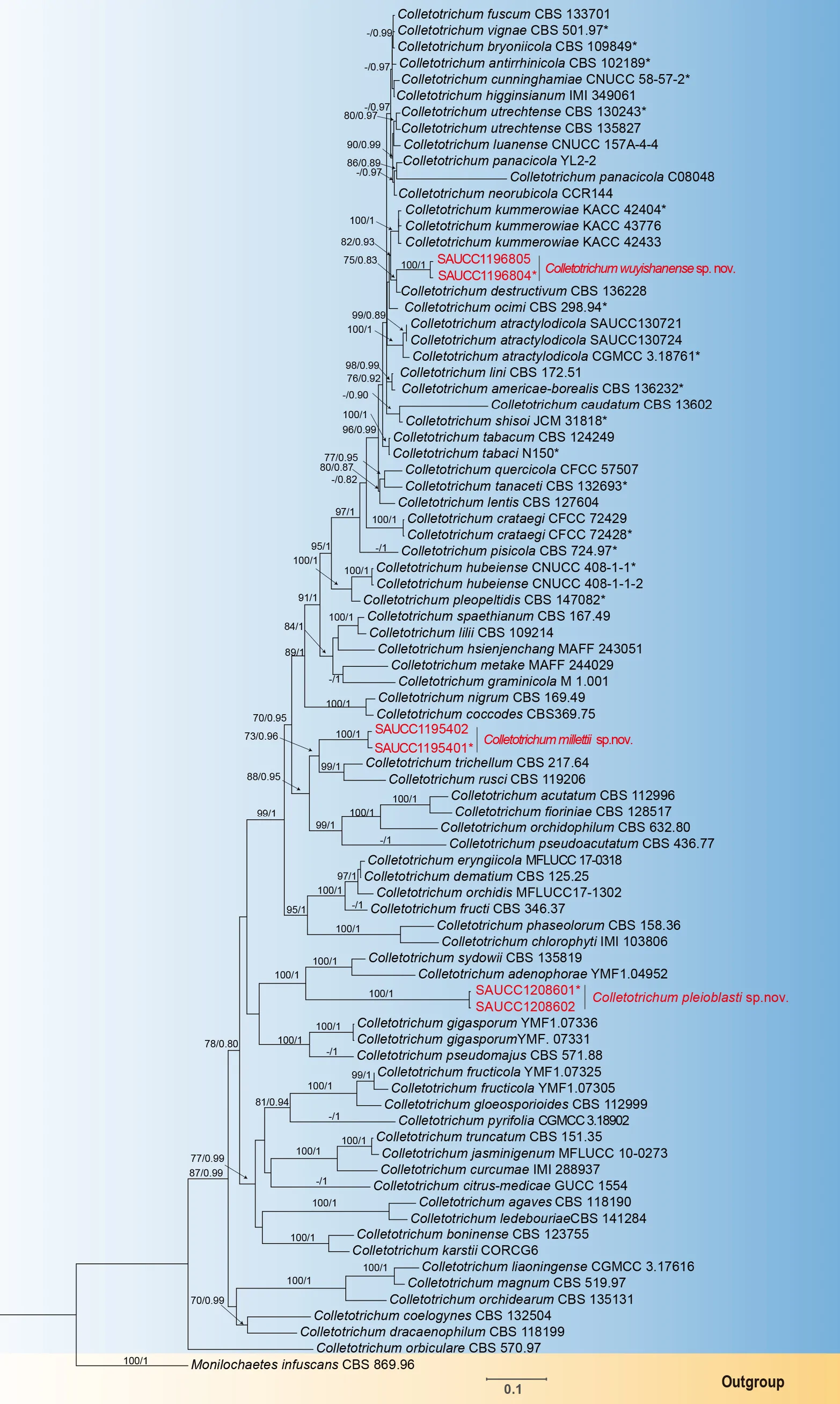
Maximum likelihood inference tree based on a combined dataset of analyzed ITS, *gapdh*, *chs-1*, *act*, and *tub2* sequences. MLBS values (left, MLBS ≥ 70%) and BYPP values (right, BYPP ≥ 0.80) are shown as MLBS/BYPP above the nodes. Strains marked with “*” are ex-type or ex-epitype strains. The text “*Colletotrichum*” is shown in a blue box and “outgroup” in a yellow box. Strains isolated in this study are indicated in red. The scale bar at the bottom indicates 0.1 substitutions per site.

Phylogenetic inference based on the concatenated ITS, *gapdh*, *chs-1*, *act*, and *tub2* dataset yielded a tree comprising 84 sequences. Six of these sequences were derived from strains isolated in the present study, and the remaining sequences were obtained from GenBank. *Monilochaetes infuscans* (CBS 869.96) served as the outgroup. This tree focuses on taxa closely allied to the isolates obtained in this study and does not encompass all known species groups within the genus *Colletotrichum*. A total of 2,271 characters, including gaps, were included in the phylogenetic analysis: 1–643 for ITS, 644–1,064 for *gapdh*, 1,065–1,349 for *chs-1*, 1,350–1,661 for *act*, and 1,662–2,271 for *tub2*. Of these, 1,036 characters were constant, 239 variable characters were parsimony-uninformative, and 996 characters were parsimony-informative. In the Bayesian analysis, the GTR+I+G model was selected for ITS, *chs-1*, and *tub2*, and the HKY+I+G model for *gapdh* and *act*. Phylogenetic trees generated using the ML method and BI algorithm shared similar topologies; thus, they can be considered to represent the evolutionary history of *Colletotrichum*. The best-scoring ML evolutionary tree, in which ML bootstrap values and Bayesian posterior probabilities are labeled at the nodes, is shown in Fig. 1. In the phylogeny, the six isolates obtained in this study formed three independent, highly supported monophyletic clades that were clearly separated from all previously described *Colletotrichum* taxa. Each clade included two isolates, and pronounced genetic divergence among the clades supports the delimitation of these three lineages as three species of *Colletotrichum*. In terms of phylogenetic affinity, *Colletotrichum millettii* (SAUCC1195401 and SAUCC1195402) was closely related to *C. trichellum* (CBS 217.64) and *C. rusci* (CBS 119206), with support values of MLBS/BYPP = 73/0.96. *Colletotrichum pleioblasti* (SAUCC1208601 and SAUCC1208602) was closely related to *C. adenophorae* (YMF1.04952) and *C. sydowii* (CBS135819), with support values of MLBS/BYPP = 100/1.00. *Colletotrichum wuyishanense* (SAUCC1196804 and SAUCC1196805) was closely related to *C. destructivum* (CBS 136228), with support values of MLBS/BYPP = 75/0.83.

**Figure 2.**
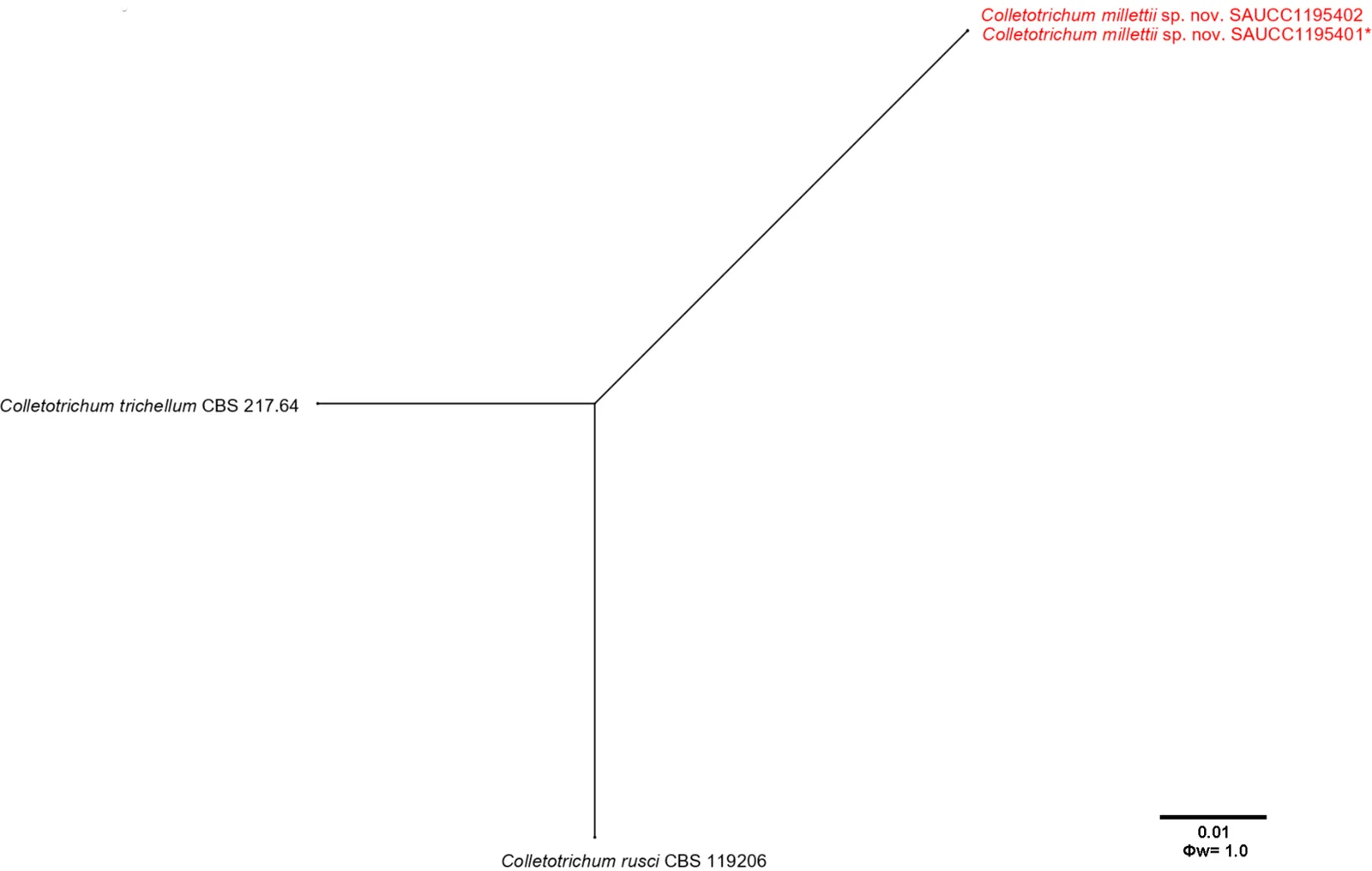
Split graph displaying the results of the pairwise homoplasy index (PHI) test of *Colletotrichum millettii* sp. nov. and closely related taxa using Log-det transformation and splits decomposition options. *Φw* ≥ 0.05 from the PHI test signifies no substantial recombination in the dataset. Type strains are marked with “*,” and the newly identified taxon and isolates are displayed in red.

To further validate the taxonomic independence of the novel taxa, the GCPSR criterion was applied. The PHI test was used to detect recombination events among closely related taxa. A *Φw* value below 0.05 indicates significant recombination, whereas values above this threshold support genetic separation. For *C. millettii* and *C. pleioblasti*, the PHI test returned non-significant values (*Φw* = 1.0), indicating no detectable recombination with closely related species, including *C. trichellum*, *C. rusci*, *C. adenophorae*, and *C. sydowii* (Figs 2, 4). For *C. wuyishanense*, the PHI test revealed no significant recombination with closely related reference species (*Φw* = 0.3201), including *C. destructivum*, *C. kummerowiae*, and *C. ocimi* (Fig. 6). These results support the recognition of *C. millettii*, *C. pleioblasti*, and *C. wuyishanense* as phylogenetically and evolutionarily distinct species.

### Taxonomy

#### Colletotrichum millettii

Taxon classificationFungiGlomerellalesGlomerellaceae

X.G. Yan, S. Wang & X.G. Zhang sp. nov.

8DCB7510-18D0-5D20-A134-15A453FDF5B0

Fungal Names: FN 573969

Figs 2, 3

##### Type.

China • Fujian Province: Wuyishan National Park, on diseased leaves of *Millettia rugosa*, 20 October 2024, X.G. Yan (HMAS 354634, holotype), ex-type living culture SAUCC1195401 = SAUCC1195402.

##### Etymology.

Referring to the genus name of the host plant *Millettia rugosa*.

##### Description.

Associated with diseased leaves of *Millettia rugosa*. Asexual morph: Conidiomata black, relatively tough, releasing conidia in pale yellow masses. Conidiophores indistinct, often reduced to conidiogenous cells. Conidiogenous cells monoblastic, hyaline, smooth, subcylindrical, slightly tapered toward the conidial end, 7.5–13.3 × 1.4–3.0 μm (x̄ = 14.1 × 3.6 μm, *n* = 40). Conidia hyaline, smooth-walled, cylindrical, straight or slightly curved, containing 1–2 large guttules, 9.7–16.4 × 2.9–4.4 μm (x̄ = 11.1 × 2.1 μm, *n* = 60). Appressoria not observed. Chlamydospores and setae not observed. Sexual morph: Undetermined.

##### Culture characteristics.

Colonies on PDA grow rapidly and are circular to subcircular. After 14 days of dark incubation at 25 °C, colonies reach 71–80 mm diameter with a growth rate of 5.1–5.7 mm/day. Dense cottony to felty aerial mycelium is white to pale gray; the center is thicker and darker, fading to pale loose mycelium at the regular entire margin. Abundant white granular acervuli scatter radially across central and subcentral zones. The reverse displays dark gray-green to dark brown centers that fade to pale white margins with distinct radial and concentric patterns. No soluble pigments are secreted into transparent medium, and central reverse pigmentation deepens from dense mycelia and conidial masses. Sparse sporulation was observed on PDA after 14 days of incubation.

##### Notes.

Based on phylogenetic analysis of combined sequence data from five loci (ITS, *gapdh*, *chs-1*, *act*, and *tub2*), *Colletotrichum millettii* (SAUCC1195401 and SAUCC1195402) constitutes an independent clade in the phylogenetic trees, closely related to *C. trichellum* (CBS 217.64) and *C. rusci* (CBS 119206). *Colletotrichum millettii* (SAUCC1195401, ex-type) is distinguished from *C. trichellum* (CBS 217.64) by 15/525, 19/268, 13/251, 21/233, and 42/413 characters in ITS, *gapdh*, *chs-1*, *act*, and *tub2* sequences, respectively, with all gap sites excluded from the sequence comparisons. *Colletotrichum millettii* (SAUCC1195401, ex-type) is distinguished from *C. rusci* (CBS 119206) by 19/529, 13/211, 16/251, 29/232, and 47/413 characters in ITS, *gapdh*, *chs-1*, *act*, and *tub2* sequences, respectively, with all gap sites excluded from the sequence comparisons. Morphologically, the conidia of *C. millettii* are shorter than those of *C. trichellum* and *C. rusci* (9.7–16.4 × 2.9–4.4 μm vs. 16–22 × 3 μm vs. 17.5–21 × 4–4.5 μm). The conidiogenous cells of *C. millettii* are longer than those of *C. rusci* (7.5–13.3 × 1.4–3.0 μm vs. 6–8 × 3–4 μm). Moreover, the PHI test showed no significant evidence of recombination between *C. millettii* and its closest relatives, *C. trichellum* and *C. rusci* (*Φw* = 1.0; Fig. 2), further supporting its status as a distinct lineage. In conclusion, robust phylogenetic evidence, unique morphological features, and marked genetic divergence justify describing *C. millettii* as a new singleton species.

**Figure 3.**
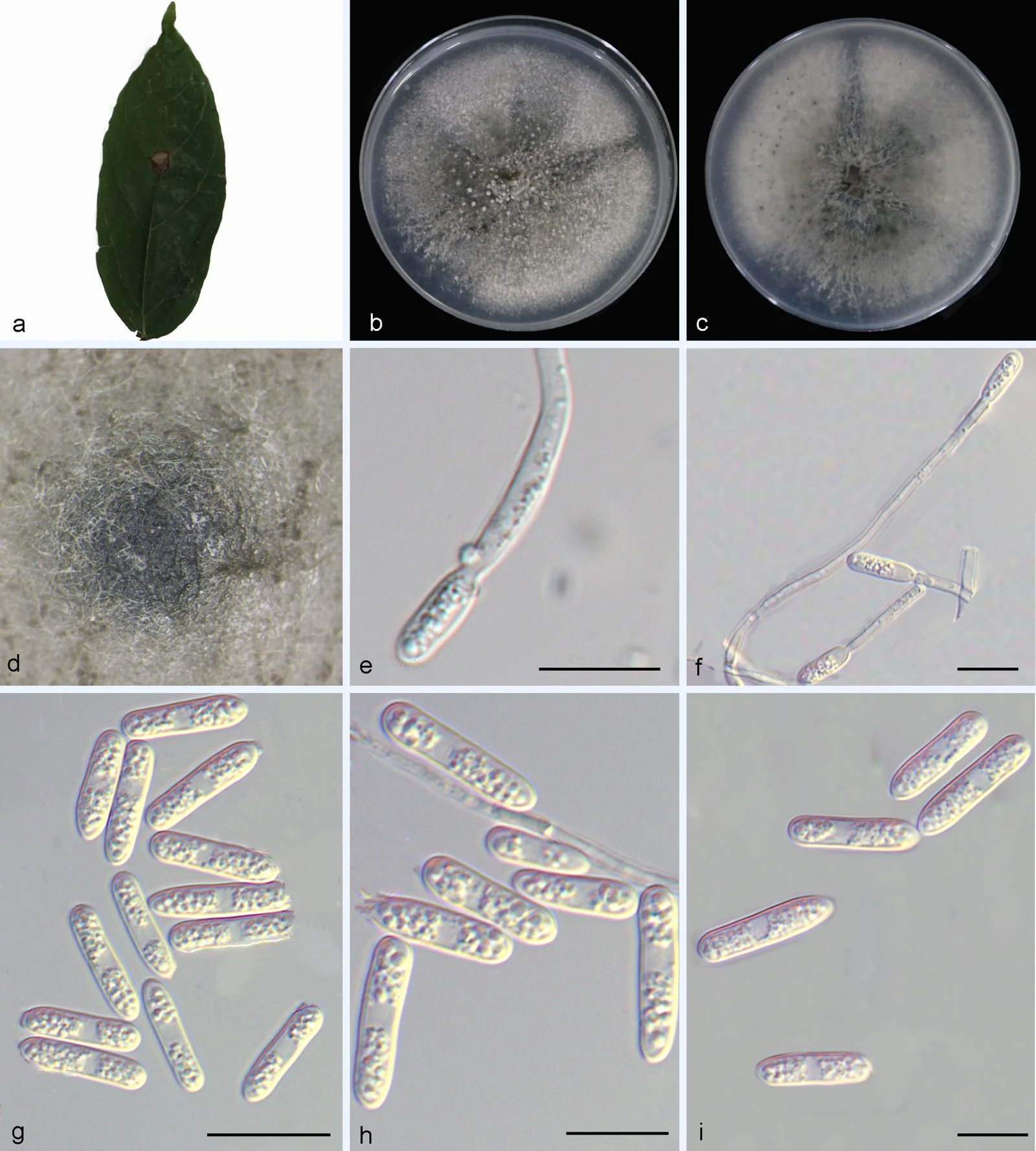
*Colletotrichum millettii* (SAUCC1195401, ex-type). **a**. A diseased leaf of *Millettia rugosa*; **b, c**. Colony on PDA from above and below after 14 days; **d**. Colony overview with conidiomata; **e, f**. Conidiogenous cells with conidia; **g–i**. Conidia. Scale bars: 10 μm (**e–i**).

#### Colletotrichum pleioblasti

Taxon classificationFungiGlomerellalesGlomerellaceae

X.G. Yan, S. Wang & X.G. Zhang sp. nov.

A91C31C1-C6D7-5D5B-9BBF-9023B61FD5A9

Fungal Names: FN 573970

Figs 4, 5

##### Type.

China • Fujian Province: Sanming City, on diseased leaves of *Pleioblastus fortunei*, 23 October 2024, X.G. Yan (HMAS 354636, holotype), ex-type living culture SAUCC1208601 = SAUCC1208602.

##### Etymology.

Referring to the genus name of the host plant *Pleioblastus*.

##### Description.

Associated with diseased leaves of *Pleioblastus fortunei*. Asexual morph: Conidiomata black, hard, producing conidia aggregated into yellow masses. Conidiophores generally not visible, reduced to conidiogenous cells. Conidiogenous cells hyaline, smooth-walled, generally cylindrical, containing an unknown substance, 20.2–47.7 × 4.1–5.7 μm (x̄ = 34.4 × 5.0 μm, *n* = 30). Conidia hyaline, aseptate, smooth-walled, cylindrical to slightly clavate, with rounded apices and truncate to slightly rounded bases, guttulate, containing numerous small, scattered oil droplets, 26.5–32.7 × 7.2–10.7 μm (x̄ = 28.7 × 9.2 μm, *n* = 50). Appressoria not observed. Chlamydospores and setae not observed. Sexual morph: Undetermined.

##### Culture characteristics.

On PDA, colonies circular to subcircular, fast-growing, reaching 71–80 mm diam. after 14 days dark incubation at 25 °C (5.1–5.7 mm/day). Aerial mycelium dense, cottony-felty, white to pale gray, thicker and darker centrally, paler and looser at margins. Numerous white granular acervuli radiate across central and subcentral regions; margin entire and smooth. Reverse dark gray-green to dark brown at the center, fading pale white outward with obvious radial/concentric color zones; medium lacking diffusible pigments and transparent whitish. Reverse shows radiating mycelial texture, darkest in the center from dense mycelium and conidiomata, pale and sparse at the margin. Sporulation commenced on PDA after 14 days of incubation.

##### Notes.

**Figure 4.**
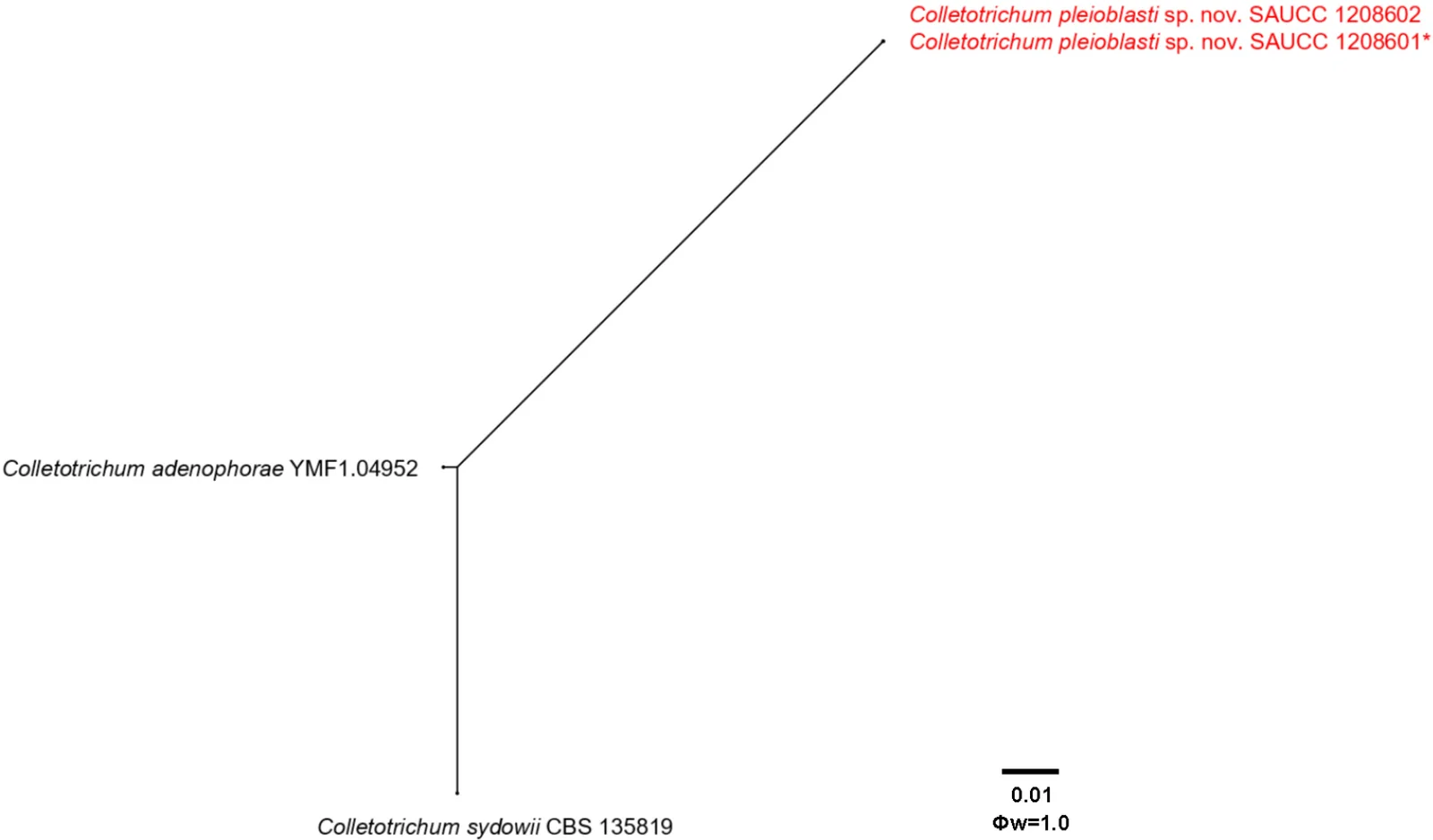
Split graph displaying the results of the pairwise homoplasy index (PHI) test of *Colletotrichum pleioblasti* sp. nov. and closely related taxa using Log-det transformation and splits decomposition options. *Φw* ≥ 0.05 from the PHI test signifies no substantial recombination in the dataset. Type strains are marked with “*,” and the newly identified taxon and isolates are displayed in red.

Based on phylogenetic analysis of combined sequence data from five loci (ITS, *gapdh*, *chs-1*, *act*, and *tub2*), *Colletotrichum pleioblasti* constitutes an independent clade in the phylogenetic trees, closely related to *C. adenophorae* (YMF1.04952) and *C. sydowii* (CBS135819). *Colletotrichum pleioblasti* (SAUCC1208601, ex-type) is distinguished from *C. adenophorae* (YMF1.04952) by 17/523, 24/252, and 19/228 characters in ITS, *gapdh*, and *chs-1* sequences, respectively, excluding gap sites. *Colletotrichum pleioblasti* (SAUCC1208601, ex-type) is distinguished from *C. sydowii* (CBS135819) by 17/498, 41/277, 21/280, 36/269, and 73/473 characters in ITS, *gapdh*, *chs-1*, *act*, and *tub2* sequences, respectively, excluding gap sites. Morphologically, the conidia of *C. pleioblasti* are longer than those of *C. adenophorae* and *C. sydowii* (26.5–32.7 × 7.2–10.7 μm vs. 18–22.5 × 5.5–7.5 μm vs. 15.5–18.5 × 5–6 μm). Moreover, the PHI test showed no significant evidence of recombination between *C. pleioblasti* and its closest relatives, *C. adenophorae* and *C. sydowii* (*Φw* = 1.0; Fig. 4), further supporting its status as a distinct lineage. In conclusion, robust phylogenetic evidence, unique morphological features, and marked genetic divergence justify describing *C. pleioblasti* as a new singleton species.

**Figure 5.**
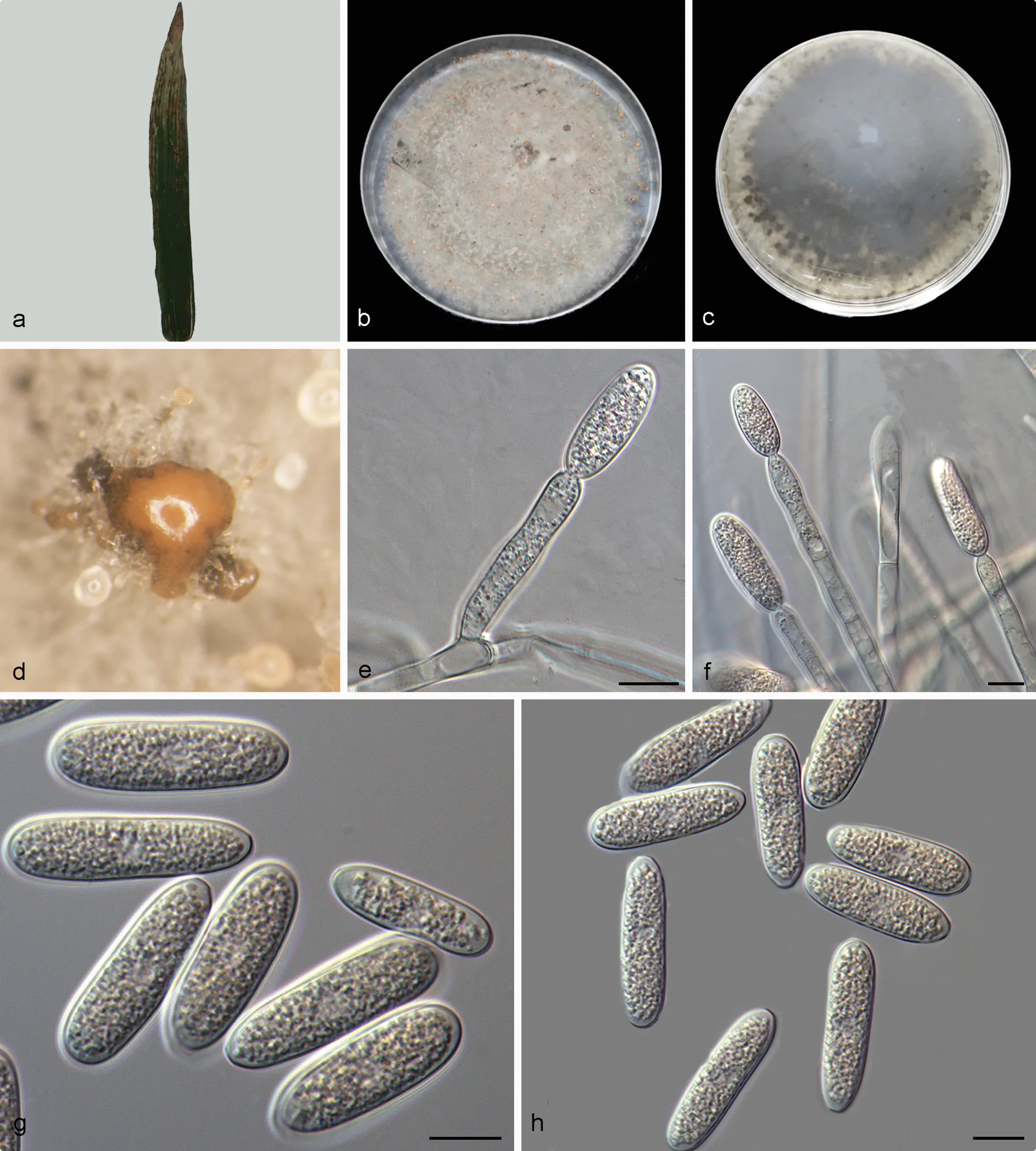
*Colletotrichum pleioblasti* (SAUCC1208601, ex-type). **a**. A diseased leaf of *Pleioblastus fortunei*; **b, c**. Colony on PDA from above and below after 14 days; **d**. Colony overview with conidiomata; **e, f**. Conidiogenous cells with conidia; **g, h**. Conidia. Scale bars: 10 μm (**e–h**).

#### Colletotrichum wuyishanense

Taxon classificationFungiGlomerellalesGlomerellaceae

X.G. Yan, S. Wang & X.G. Zhang sp. nov.

F9F2599B-33F2-5705-A97B-B5E41E35BF4F

Fungal Names: FN 573971

Figs 6, 7

##### Type.

China • Fujian Province: Wuyishan National Park, on diseased leaves of *Paederia scandens*, 20 October 2024, X.G. Yan (HMAS 354635, holotype), ex-type living culture SAUCC1196804 = SAUCC1196805.

##### Etymology.

Referring to the type locality, Wuyishan National Park.

##### Description.

Associated with diseased leaves of *Paederia scandens*. Asexual morph: Conidiomata black, hard, producing conidia aggregated into pale brown masses. Conidiophores hyaline, straight or slightly curved, 5.8–10.4 × 2.1–4.2 μm (x̄ = 7.8 × 2.8 μm, *n* = 25). Conidiogenous cells cylindrical, rounded at both ends, hyaline, smooth-walled, 3.5–5.6 × 1.3–3.3 μm (x̄ = 4.8 × 2.1 μm, *n* = 25). Conidia hyaline, smooth, ellipsoidal, containing 1–3 large guttules, 11.2–16.1 × 5.7–7.4 μm (x̄ = 14.1 × 6.3 μm, *n* = 50). Appressoria black, dome-shaped, up to 9 μm in length. Chlamydospores and setae not observed. Sexual morph: Undetermined.

##### Culture characteristics.

Colonies circular and moderately growing on PDA, distinctly zonate after 14 days dark incubation at 25 °C. Aerial mycelium abundant, cottony, pure white overall; central colony compact with pale gray-brown to dark fuscous discoloration, fading to lose white mycelium near the margin. Small granular conidiomata sparsely scattered in the median area; margin undulate, with tiny satellite microcolonies emerging peripherally. Reverse dark brown to black at the center, gradually pale cream-white outward, showing clear concentric rings. No diffusible pigments produced in agar. Sporulation commenced on PDA after 21 days of incubation.

##### Notes.

**Figure 6.**
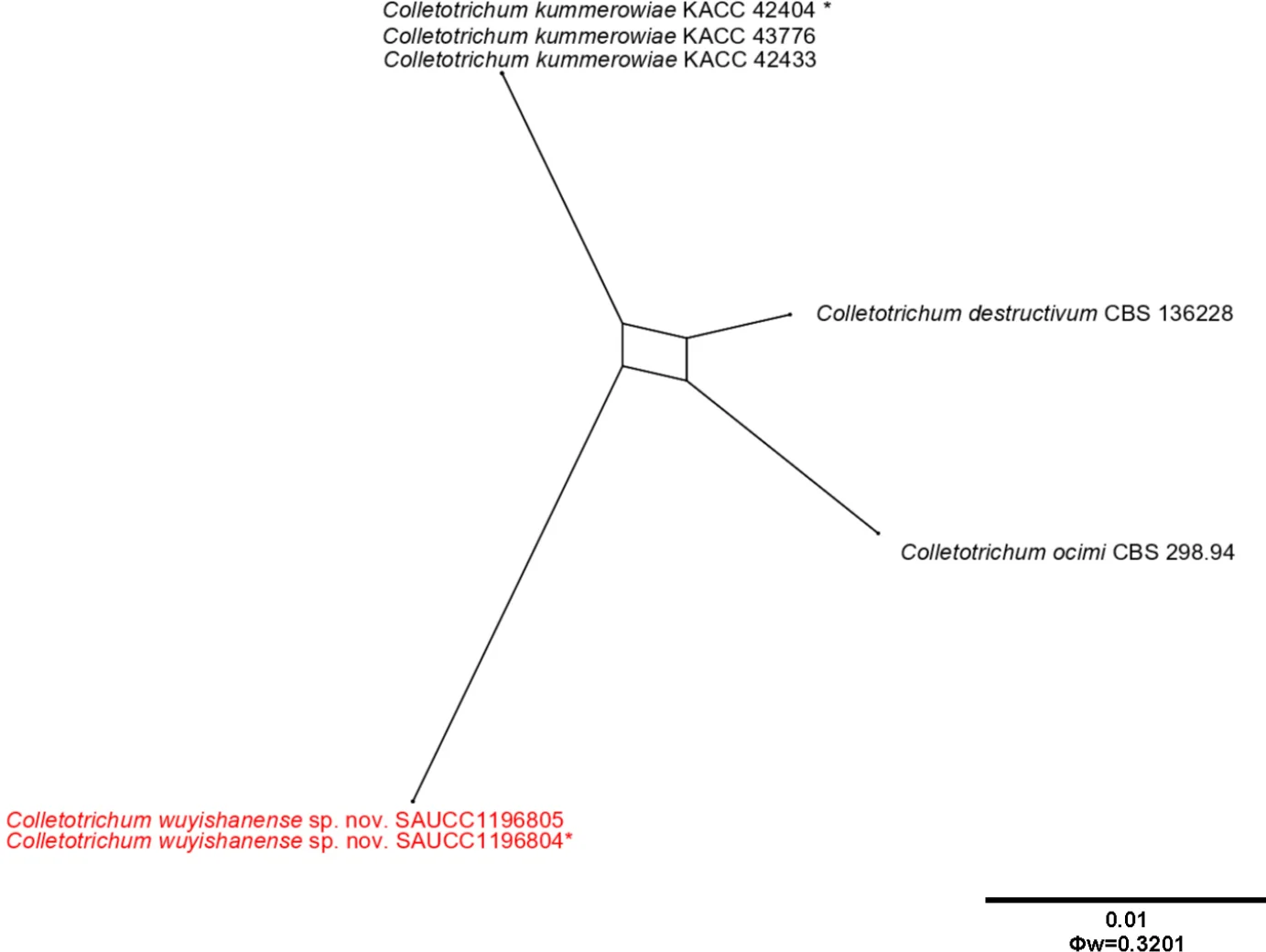
Split graph displaying the results of the pairwise homoplasy index (PHI) test of *Colletotrichum wuyishanense* sp. nov. and closely related taxa using Log-det transformation and splits decomposition options. *Φw* ≥ 0.05 from the PHI test signifies no substantial recombination in the dataset. Type strains are marked with “*,” and the newly identified taxon and isolates are displayed in red.

Based on phylogenetic analysis of combined sequence data from five loci (ITS, *gapdh*, *chs-1*, *act*, and *tub2*), *Colletotrichum wuyishanense* (SAUCC1196804 and SAUCC1196805) constitutes an independent clade in the phylogenetic trees, closely related to *C. destructivum* (CBS 136228) and *C. kummerowiae* (KACC 42404, KACC 42433, and KACC 43776). *Colletotrichum wuyishanense* (SAUCC1196804, ex-type) is distinguished from *C. destructivum* (CBS 136228) by 13/486, 6/191, 7/285, and 21/266 characters in ITS, *gapdh*, *chs-1*, and *act* sequences, respectively, excluding gap sites. *Colletotrichum wuyishanense* (SAUCC1196804, ex-type) is distinguished from *C. kummerowiae* (KACC 42404, ex-type) by 12/486, 7/149, 3/251, and 16/246 characters in ITS, *gapdh*, *chs-1*, and *act* sequences, respectively, excluding gap sites. Morphologically, the conidiogenous cells of *C. wuyishanense* are shorter than those of *C. destructivum* and *C. kummerowiae* (3.5–5.6 × 1.3–3.3 μm vs. 9.5–17 × 3.5–5 μm vs. 8–18 × 3.5–4.5 μm). The conidia of *C. wuyishanense* are shorter than those of *C. destructivum* and *C. kummerowiae* (11.2–16.1 × 5.7–7.4 μm vs. 14–22 × 3.5–5 μm vs. 15.5–18.5 × 3.5–4.5 μm). Moreover, the PHI test showed no significant evidence of recombination between *C. wuyishanense* and its closest relatives, *C. destructivum* and *C. kummerowiae* (*Φw* = 0.3201; Fig. 6), further supporting its status as a distinct lineage. In conclusion, the combination of strong phylogenetic support, clear morphological differentiation, and genetic divergence justifies the recognition of *C. wuyishanense* as a novel species within the *C. destructivum* species complex.

**Figure 7.**
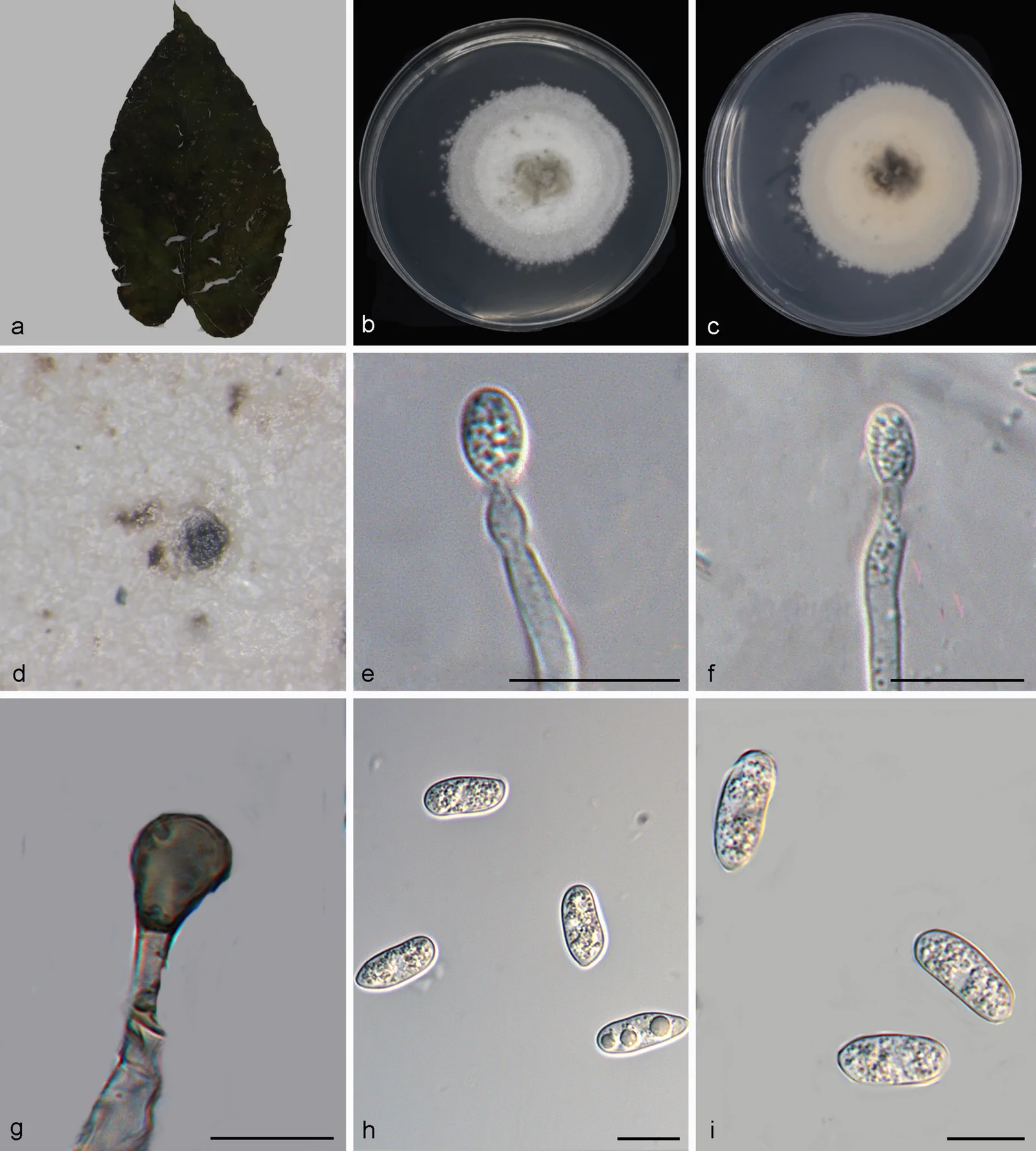
*Colletotrichum wuyishanense* (SAUCC1196804, ex-type). **a**. A diseased leaf of *Paederia scandens*; **b, c**. Colony on PDA from above and below after 14 days; **d**. Colony overview with conidiomata; **e, f**. Conidiogenous cells with conidia; **g**. Appressorium; **h, i**. Conidia. Scale bars: 10 μm (**e–i**).

## Discussion

*Colletotrichum* is a globally prevalent genus of phytopathogenic fungi, notorious for causing anthracnose diseases on a wide range of vascular plants. Multilocus phylogenetic analyses based on GCPSR criteria have greatly refined the taxonomic framework of this genus, revealing numerous cryptic species embedded in well-defined species complexes (Taylor et al. 2000; Liu et al. 2022). In China, previous studies on *Colletotrichum* have covered both economic crops and wild plants across tropical and subtropical regions. In the present study, three novel *Colletotrichum* species were isolated from diseased leaves of wild plant species in Fujian, China. Integrated molecular, morphological, and cultural evidence robustly validates these three taxa as new species.

Phylogenetic analyses based on five-locus (ITS, *gapdh*, *act*, *chs-1*, and *tub2*) concatenated datasets, with *Monilochaetes infuscans* as the outgroup, resolved three supported monophyletic clades (MLBS/BYPP = 100/1) corresponding to the three new species. Each taxon exhibited considerable genetic divergence from all known *Colletotrichum* species, supporting species delimitation under the GCPSR criterion. The novel species *Colletotrichum wuyishanense* clustered within the *C. destructivum* species complex, forming a unique subclade clearly separated from closely related species such as *C. destructivum* and *C. higginsianum*. In contrast, *Colletotrichum millettii* and *C. pleioblasti* each formed distinct, standalone monophyletic lineages within the genus. Although they are phylogenetically clustered closely with singleton species such as *C. rusci*, they could not be assigned to any previously described *Colletotrichum* species complex, representing previously unrecognized evolutionary branches of the genus. All three new species exhibit typical morphological features diagnostic of the genus *Colletotrichum*. Notably, appressorium formation, a key phenotypic character of *Colletotrichum*, was observed in *C. wuyishanense*, whereas appressoria were not produced by the other two novel species under the experimental conditions. The combination of unique phylogenetic positions and stable morphological and cultural traits provides sufficient polyphasic evidence for the establishment of these new species.

Fujian’s subtropical monsoon climate provides humid and warm conditions suitable for *Colletotrichum* colonization and diversification. The three new species, *C. wuyishanense*, *C. millettii*, and *C. pleioblasti*, were isolated from three distinct native plant hosts, widening the host range of foliar anthracnose pathogens in Southeast China. The discovery of three novel lineages demonstrates that the current diversity of *Colletotrichum* in subtropical China is still underestimated and that non-crop native plants are important reservoirs of undescribed cryptic *Colletotrichum* species.

This study characterizes three novel *Colletotrichum* species. One of them, *C. wuyishanense*, increases the known species diversity of the *C. destructivum* species complex, whereas the other two new taxa, *C. millettii* and *C. pleioblasti*, represent previously undocumented lineages that extend understanding of phylogenetic variation within the genus *Colletotrichum*. The generated molecular barcodes and ex-type strains serve as valuable resources for future taxonomic research. As pathogenicity validation was not performed in this study, future work will include pathogenicity tests and host-range assays, together with systematic field investigations, to clarify the pathogenic traits, host specificity, and distribution patterns of these novel taxa.

## Conclusion

Samples of diseased leaves were collected from Fujian Province, China. Multiple strains of the genus *Colletotrichum* were isolated using tissue isolation and single-spore purification techniques. Three new species of *Colletotrichum*, *C. wuyishanense*, *C. millettii*, and *C. pleioblasti*, were identified based on morphological observations and multilocus phylogenetic analysis. Among them, *C. wuyishanense* falls within the *C. destructivum* species complex. By contrast, *C. millettii* and *C. pleioblasti* form two distinct monophyletic lineages nested within the genus *Colletotrichum* yet cannot be assigned to any formally described species complex. Additional novel and unrecorded species of *Colletotrichum* are likely to be discovered from subtropical plant hosts across China, further enriching the documented species diversity of this genus and providing fundamental taxonomic and ecological evidence for native plant conservation and regional anthracnose management.

## Supplementary Material

XML Treatment for Colletotrichum millettii

XML Treatment for Colletotrichum pleioblasti

XML Treatment for Colletotrichum wuyishanense

## Additional information

### Artificial Intelligence (AI) use

The authors accept full responsibility for the content of the manuscript, including the disclosure of any use of AI.

No AI tools were used in the preparation of this manuscript.

### Author ORCIDs

X.-G. Yan https://orcid.org/0009-0005-6257-3490

Y. Jiang https://orcid.org/0009-0003-1292-610X

X.-S. Wang https://orcid.org/0009-0005-1127-7368

J.-Y. Sheng https://orcid.org/0009-0003-3882-6748

Y. Zhang https://orcid.org/0009-0002-9239-8097

C.-C. Ai https://orcid.org/0009-0002-3934-7944

X.-G. Zhang https://orcid.org/0000-0001-9733-8494

S. Wang https://orcid.org/0000-0002-7376-7638

### Data availability

All of the data that support the findings of this study are available in the main text or Supplementary Information.

## Supplementary materials

Supplementary material 1 GenBank accession numbers of the sequences used in phylogenetic analyses in this study analyses in this study Data type docx https://binary.pensoft.net/file/1754449This dataset is made available under the Open Database License (http://opendatacommons.org/licenses/odbl/1.0/). The Open Database License (ODbL) is a license agreement intended to allow users to freely share, modify, and use this Dataset while maintaining this same freedom for others, provided that the original source and author(s) are credited. Xi-Gang Yan, Yang Jiang, Xing-Sheng Wang, Jin-Yan Sheng, Yue Zhang, Cong-Cong Ai, Xiu-Guo Zhang, Shi Wang
