# Supplementary material for: Three new species of Colletotrichum (Glomerellaceae, Glomerellales) based on morphology and multilocus phylogeny from Fujian, China
Source: MycoKeys. 2026 Aug 25;139:139–55. doi: 10.3897/mycokeys.139.208158 (PMC13535500; doi:10.3897/mycokeys.139.208158)
Supplement: Supplementary file 1 [file mycokeys-139-139-s001.docx]

| Species  **Table S1** GenBank accession numbers of the sequences used in phylogenetic analyses in this study analyses in this study. | Strain | Locality | Substrate | ITS | *gapdh* | *chs-1* | *act* | *tub2* | References |
| --- | --- | --- | --- | --- | --- | --- | --- | --- | --- |
| *Colletotrichum acutatum* | CBS 112996 | Australia | *Grevillea* sp*.* | JQ005776 | JQ948677 | JQ005797 | JQ005839 | JQ005860 | Damm et al. (2012) |
| *C. adenophorae* | YMF1.04952 | China | *Ageratina adenophora* | OK030859 | OK513662 | OK513558 | - | - | Yu et al. (2022) |
| *C. agaves* | CBS 118190 | Mexico, Netherlands | *Agave* spp. | DQ286221 | - | - | - | - | Farr et al. (2006) |
| *C. americae-borealis* | CBS 136232* | USA | *Medicago sativa* | KM105224 | KM105579 | KM105294 | KM105434 | KM105504 | Damm et al. (2014) |
| *C. antirrhinicola* | CBS 102189* | New Zealand | *Antirrhinum majus* | KM105180 | KM105531 | KM105250 | KM105390 | KM105460 | Damm et al. (2014) |
| *C. atractylodicola* | CGMCC 3.18761* | China | *Atractylodes chinensis* | KR149280 | KR259334 | KR259333 | KR132243 | KU058178 | Xu et al. (2018) |
| *C. atractylodicola* | SAUCC130721 | China | *Atractylodes chinensis* | KU289190 | KU289205 | KU289200 | KU289195 | KU289210 | Xu et al. (2018) |
| *C. atractylodicola* | SAUCC130724 | China | *Atractylodes chinensis* | KU289191 | KU289206 | KU289201 | KU289196 | KU289211 | Xu et al. (2018) |
| *C. boninense* | CBS 123755 | Worldwide | *Annonaceae* | JQ005153 | JQ005240 | JQ005327 | JQ005501 | JQ005588 | Damm et al. (2012) |
| *C. bryoniicola* | CBS 109849* | Netherlands | *Bryonia dioica* | KM105181 | KM105532 | KM105251 | KM105391 | KM105461 | Damm et al. (2014) |
| *C. caudatum* | CBS 131602 | USA | *Sorghastrumnutans* | JX076860 | - | - | - | - | New York State Museum. (1909) |
| *C. chlorophyti* | IMI 103806 | Australia, India | *Chlorophytum* sp. | GU227894 | GU228286 | GU228384 | GU227992 | GU228188 | Damm et al. (2009) |
| *C. citrus-medicae* | GUCC 1554 | China | *Citrus medica* | MN959910 | MT006331 | MT006328 | MT006325 | - | Hyde et al. (2020) |
| *C. coccodes* | CBS 369.75 | The Netherlands | *Rubus coreanus* | HM171679 | HM171673 | JQ005796 | HM171667 | JQ005859 | From NCBI |
| *C. coelogynes* | CBS 132504 | Germany | *Coelogyne* sp. | MG600713 | MG600776 | MG600836 | MG600920 | MG600980 | Damm et al. (2019) |
| *C. crataegi* | CFCC 72428 T | China | *Crataegus pinnatifida* | PV524701 | PV573491 | PV573523 | PV573552 | PV573583 | Zhang and Fan (2025) |
| *C. crataegi* | CFCC 72429 | China | *Crataegus pinnatifida* | PV524702 | PV573492 | PV573524 | PV573553 | PV573584 | Zhang and Fan (2025) |
| *C. cunninghamiae* | CNUCC 58-57-2* | China | *Cunninghamia lanceolata* | PP812213 | PP831925 | PP820670 | PP820664 | PP831937 | Sui et al. (2024) |
| *C. curcumae* | IMI 288937 | India | *Curcuma longa* | GU227893 | GU228285 | GU228383 | GU227991 | GU228187 | Damm et al. (2009) |
| *C. dematium* | CBS 125.25 | France | *Eryngium campestre* | GU227819 | GU228211 | GU228309 | GU227917 | GU228113 | Damm et al. (2009) |
| *C. destructivum* | CBS 136228 | USA | *Trifolium hybridum* | KM105207 | KM105561 | KM105277 | KM105417 | KM105487 | Damm et al. (2014) |
| *C. dracaenophilum* | CBS 118199 | Bulgaria | *Dracaena* sp. | JX519222 | JX546707 | JX519230 | JX519238 | JX519247 | Cannon et al. (2012) |
| *C. eryngiicola* | MFLUCC 17-0318 | Russia | *Eryngium campestre* | KY792726 | KY792723 | KY792720 | KY792717 | KY792729 | Buyck et al. (2017) |
| *C. fioriniae* | CBS 128517 | Australia, Netherlands, UK, USA | *Magnifera indica* (stem endophyte) | JQ948292 | JQ948622 | JQ948953 | JQ949613 | JQ949943 | Damm et al. (2012) |
| *C. fructi* | CBS 346.37 | USA | *Malus sylvestris* | GU227844 | GU228236 | GU228334 | GU227942 | GU228138 | Damm et al. (2009) |
| *C. fructicola* | YMF1.07305 | China | *Ageratinaadenophora* | OK030893 | OK513694 | OK513590 | OK513624 | OK513655 | Cai et al. (2009) |
| *C. fructicola* | YMF1.07325 | China | *Ageratinaadenophora* | OK030892 | OK513693 | OK513589 | OK513623 | OK513654 | Cai et al. (2009) |
| *C. fuscum* | CBS 133701 | Germany | *Digitalis lanata* | KM105174 | KM105524 | KM105244 | KM105384 | KM105454 | Damm et al. (2014) |
| *C. gigasporum* | YMF1.07331 | China | *Ageratinaadenophora* | OK030895 | OK513696 | OK513592 | OK513626 | OK513657 | Rakotoniriana et al. (2013) |
| *C. gigasporum* | YMF1.07336 | China | *Ageratinaadenophora* | OK030894 | OK513695 | OK513591 | OK513625 | OK513656 | Rakotoniriana et al. (2013) |
| *C. gloeosporioides* | CBS 112999 | Australia | *Citrus* sp. | JQ005152 | JQ005239 | JQ005326 | JQ005500 | JQ005587 | Damm et al. (2012b) |
| *C. graminicola* | M 1.001/CBS 130836 | Worldwide | *Zea mays* | JQ005767 | - | JQ005788 | JQ005830 | JQ005851 | O’Connell et al. (2012) |
| *C. higginsianum* | IMI 349061 | Trinidad | *Brassica chinensis* | KM105184 | KM105535 | KM105254 | KM105394 | KM105464 | Damm et al. (2014) |
| *C. hsienjenchang* | MAFF 243051 | Japan | *Phyllostachys* sp. | AB738855 | - | AB738846 | AB738845 | - | From NCBI |
| *C. hubeiense* | CNUCC 408-1-1* | China | *Lindera glauca* | PP812212 | PP831924 | PP820669 | PP820663 | PP831936 | Sui et al. (2024) |
| *C. hubeiense* | CNUCC 408-1-1- 2 | China | *Lindera glauca* | PP812216 | PP831928 | PP820673 | PP820667 | PP831940 | Sui et al. (2024) |
| *C. jasminigenum* | MFLUCC 10- 0273 | Vietnam | *Jasminum sambac* | HM131513 | - | - | - | HM153770 | Wikee et al. (2011) |
| *C. karstii* | CORCG6 | China | *Annona cherimola* | HM585409 | HM585391 | HM582023 | HM581995 | HM585428 | Yang et al. (2011) |
| *C. kummerowiae* | KACC 43776 | Korea | *Kummerowia striata* | OR431672 | OR449471 | OR449445 | OR449419 | OR449501 | Thao et al. (2024) |
| *C. kummerowiae* | KACC 42433 | Korea | *Kummerowia striata* | OR431668 | OR449468 | OR449442 | OR449420 | OR449503 | Thao et al. (2024) |
| *C. kummerowiae* | KACC 42404 * | Korea | *Kummerowia striata* | OR431667 | OR449464 | OR449452 | OR449411 | OR449507 | Thao et al. (2024) |
| *C. ledebouriae* | CBS 141284 | South Africa | *Ledebouria floridunda* | KX228254 | - | - | KX228357 | - | Crous et al. (2013) |
| *C. lentis* | CBS 12760T | Canada | *Lens culinaris* | JQ005766 | KM105597 | JQ005787 | JQ005829 | JQ005850 | O’Connell et al. (2012) |
| *C. liaoningense* | CGMCC 3.17616 | Chin | *Capsicu annuum* | KP890104 | KP890135 | KP890127 | KP890097 | KP890111 | Diao et al. (2017) |
| *C. lilii* | CBS 109214 | Japan | *Lillium* sp. | GU227810 | GU228202 | GU228300 | GU227908 | GU228104 | Damm et al. (2009) |
| *C. lini* | CBS 172.51 | Netherlands | *Linum usitatissimum* | JQ005765 | KM105581 | JQ005786 | JQ005828 | JQ005849 | O’Connell et al. (2012) |
| *C. luanense* | CNUCC 157A-4-4* | China | *Camellia sinensis* | PP812211 | PP831923 | PP820668 | PP820662 | PP831935 | Sui et al. (2024) |
| *C. magnum* | CBS 519.97 | USA | *Citrullus lanatus* | MG600769 | MG600829 | MG600875 | MG600973 | MG601036 | Damm et al. (2019) |
| *C. metake* | MAFF 244029 | Japan | *Pleioblastus simoni* | AB738859 | - | - | - | - | From NCBI |
| ***C. millettii*** | **SAUCC1195401*** | **China** | ***Millettia rugosa*** | **PZ097555** | **PZ536005** | **PZ536011** | **PZ536013** | **PZ286101** | **This study** |
| ***C. millettii*** | **SAUCC1195402** | **China** | ***Millettia rugosa*** | **PZ097556** | **PZ536006** | **PZ536012** | **PZ536014** | **PZ286102** | **This study** |
| *C. neorubicola* | CCR144 | China | *Bubus idaeus* | MK529906 | MK547520 | MK547526 | MK547523 | MN186400 | Liu et al. (2020) |
| *C. nigrum* | CBS 169.49 | Argentina | *Capsicum* sp. | JX546838 | JX546742 | JX546693 | JX546646 | JX546885 | Liu et al. (2013) |
| *C. ocimi* | CBS 298.94 * | Italy | *Ocimum basilicum* | KM105222 | KM105577 | KM105292 | KM105432 | KM105502 | O’Connell et al. (2012) |
| *C. orbiculare* | CBS 570.97 | Worldwide | genera of *Cucubitaceae* | KF178466 | KF178490 | KF178515 | KF178563 | KF178587 | Damm et al. (2013) |
| *C. orchidearum* | CBS 135131 | China | *Cordyline* | MG600738 | MG600800 | MG600855 | MG600944 | MG601005 | Damm et al. (2019) |
| *C. orchidis* | MFLUCC 17-1302 | Italy | *Orchis* sp. | MK502144 | MK496857 | MK496855 | MK496853 | MK496859 | Hyde (2020) |
| *C. orchidophilum* | CBS 632.80 | Panama, UK, USA, France | *Ascocenda* sp. | JQ948151 | JQ948481 | JQ948812 | JQ949472 | JQ949802 | Damm et al. (2012b) |
| *C. panacicola* | C08048 | China | *Panax ginseng* | GU935867 | GU935847 | - | GU944757 | GU935887 | Thao et al. (2024) |
| *C. panacicola* | YL2-2 | China | *Panax ginseng* | MN685228 | MN894870 | MN894862 | MN894854 | MN894877 | Thao et al. (2024) |
| *C. phaseolorum* | CBS 158.36 | Japan | *Phaseolus* sp. | GU227897 | GU228289 | GU228387 | GU227995 | GU228191 | Damm et al. (2009) |
| C. *pisicola* | CBS 724.97* | USA | *Pisum sativum* | KM105172 | KM105522 | KM105242 | KM105382 | KM105452 | Damm et al. (2014) |
| ***C. pleioblasti*** | **SAUCC1208601*** | **China** | ***Pleioblastus fortunei*** | **PZ097551** | **PZ536009** | **PZ531276** | **PZ536017** | **PZ564428** | **This study** |
| ***C. pleioblasti*** | **SAUCC1208602** | **China** | ***Pleioblastus fortunei*** | **PZ097552** | **PZ536010** | **PZ531277** | **PZ536018** | **PZ564429** | **This study** |
| *C. pleopeltidis* | CBS 147082* | South Africa | *Pleopeltis* sp. | MW883412 | - | MW890035 | MW890024 | - | Crous et al. (2021) |
| *C. pseudoacutatum* | CBS 436.77 | Chile | *Pinus radiata* | JQ948480 | JQ948811 | JQ949141 | JQ949801 | JQ950131 | Damm et al. (2012a) |
| *C. pseudomajus* | CBS 571.88 | Taiwan | *Camellia sinensis* | KF687722 | KF687826 | KF687779 | KF687801 | KF687883 | Liu et al. (2014) |
| *C. pyrifolia* | CGMCC 3.18902 | China | *Pyrus pyrifolia* | MG748078 | MG747996 | MG747914 | MG747768 | MG748158 | Fu et al. (2019) |
| *C. quercicola* | CFCC 57507 | China | *Quercus variabilis* | ON692811 | ON755053 | ON755049 | ON755045 | ON755057 | Wang et al. (2022) |
| *C. rusci* | CBS 119206 | Italy | *Ruscus* sp. | GU227818 | GU228210 | GU228308 | GU227916 | GU228112 | Damm et al. (2009) |
| *C. shisoi* | JCM 31818 T | Japan | *Perilla frutescens* var. | MH660930 | MH660931 | MH660929 | MH660928 | MH660932 | Gan et al. (2019) |
| *C. spaethianum* | CBS 167.49 | Germany | *Hosta sieboldiana* | GU227807 | GU228199 | GU228297 | GU227905 | GU228101 | Damm et al. (2009) |
| *C. sydowii* | CBS135819 | Taiwan (China) | *Sambucus* sp. | KY263783 | KY263785 | KY263787 | KY263791 | KY263793 | Marin-Felix et al. (2017) |
| *C. tabaci* | N150* | Canada | *Crispa Nicotiana tabacum* | KM105204 | KM105557 | KM105274 | KM105414 | KM105484 | Damm et al. (2014) |
| *C. tabacum* | CBS 124249 | Madagascar | *Centella asiatica* | KM105206 | KM105560 | KM105276 | KM105416 | KM105486 | Damm et al. (2014) |
| *C. tanaceti* | CBS 132693* | Ulverstone | *Tanacetum cinerariifolium* | JX218228 | JX218243 | JX259268 | JX218238 | JX218233 | Barimani et al. (2013) |
| *C. trichellum* | CBS 217.64 | Canada, Germany | *Hedera* sp. | GU227812 | GU228204 | GU228302 | GU227910 | GU228106 | Damm et al. (2009) |
| *C. truncatum* | CBS 151.35 | Australia | *Arachis hypogaea* | GU227862 | GU228254 | GU228352 | GU227960 | GU228156 | Damm et al. (2009) |
| *C. utrechtense* | CBS 130243 T | Netherlands | *Trifolium pratense* | KM105201 | KM105554 | KM105271 | KM105411 | KM105481 | Damm et al. (2014) |
| *C. utrechtense* | CBS 135827 | Netherlands | *Trifolium pratense* | KM105202 | KM105555 | KM105272 | KM105412 | KM105482 | Damm et al. (2014) |
| *C. vignae* | CBS 501.97* | Nigeria | *Vigna unguiculata* | KM105183 | KM105534 | KM105253 | KM105393 | KM105463 | Damm et al. (2014) |
| ***C. wuyishanense*** | **SAUCC1196804*** | **China** | ***Paederia scandens*** | **PZ097553** | **PZ536007** | **PZ531274** | **PZ536015** | **-** | **This study** |
| ***C. wuyishanense*** | **SAUCC1196805** | **China** | ***Paederia scandens*** | **PZ097554** | **PZ536008** | **PZ531275** | **PZ536016** | **-** | **This study** |
| *Monilochaetes infuscans* | CBS 869.96 | District of Columbia | *Ipomoea batata* | JQ005780 | JX546612 | JQ005801 | - | JQ005864 | O’Connell et al. (2012) |

**Notes**: New species established in this study are shown in bold. Those marked “*” in the table are represented as ex-type or ex-epitype strains. “-”: Not available.

**References**

Barimani M, Pethybridge SJ, Vaghefi N, Hay FS, Taylor PWJ (2013) A new anthracnose disease of pyrethrum caused by *Colletotrichum tanaceti* sp. nov. Plant Pathology 62: 1248–1257. <https://doi.org/10.1111/ppa.12054>

Buyck B, Duhem B, Das K, Jayawardena RS, Niveiro N, Pereira OL, Prasher IB, Adhikari S, Albertó EO, Bulgakov TS, Castañeda-Ruiz RF, Hembrom ME, Hyde KD, Lewis DP, Michlig A, Nuytinck J, Parihar A, Popoff OF, Ramirez NA, Silva MD, Verma RK, Hofstetter V (2017) Fungal Biodiversity Profiles 21–30. Cryptogamie, Mycologie 38: 101–146. <https://doi.org/10.7872/crym/v38.iss1.2017.101>

Cai L, Hyde K, Taylor P, Weir B, Waller J, Abang M, Zhang JZ, Yang YL, Phoulivong S, Liu ZY (2009) A polyphasic approach for studying *Colletotrichum*. Fungal Diversity 39: 183–204. <https://www.fungaldiversity.org/fdp/sfdp/FD39-8.pdf>

Cannon PF, Damm U, Johnston PR, Weir BS (2012) *Colletotrichum* - current status and future directions. Studies in Mycology 73: 181–213. <https://doi.org/10.3114/sim0014>

Crous PW, Wingfield MJ, Guarro J, Cheewangkoon R, Van Der Bank M, Swart WJ, Stchigel AM, Cano-Lira JF, Roux J, Madrid H, Damm U, Wood AR, Shuttleworth LA, Hodges CS, Munster M, De Jesús Yáñez-Morales M, Zúñiga-Estrada L, Cruywagen EM, De Hoog GS, Silvera C, Najafzadeh J, Davison EM, Davison PJN, Barrett MD, Barrett RL, Manamgoda DS, Minnis AM, Kleczewski NM, Flory SL, Castlebury LA, Clay K, Hyde KD, Maússe-Sitoe SND, Chen S, Lechat C, Hairaud M, Lesage-Meessen L, Pawłowska J, Wilk M, Śliwińska-Wyrzychowska A, Mętrak M, Wrzosek M, Pavlic-Zupanc D, Maleme HM, Slippers B, Mac Cormack WP, Archuby DI, Grünwald NJ, Tellería MT, Dueñas M, Martín MP, Marincowitz S, De Beer ZW, Perez CA, Gené J, Marin-Felix Y, Groenewald JZ (2013) Fungal Planet description sheets: 154–213. Persoonia 31: 188–296. <https://doi.org/10.3767/003158513X675925>

Damm U, Woudenberg JHC, Cannon P, Crous P (2009) *Colletotrichum* species with curved conidia from herbaceous hosts. Fungal Diversity 39: 45–87. https://www.fungaldiversity.org/fdp/sfdp/FD39-3.pdf

Damm U, Cannon PF, Woudenberg JHC, Crous PW (2012a) The *Colletotrichum acutatum* species complex. Studies in Mycology 73: 37–113. <https://doi.org/10.3114/sim0010>

Damm U, Cannon PF, Woudenberg JHC, Johnston PR, Weir BS, Tan YP, Shivas RG, Crous PW (2012b) The *Colletotrichum boninense* species complex. Studies in Mycology 73: 1–36. <https://doi.org/10.3114/sim0002>

Damm U, Cannon PF, Liu F, Barreto RW, Guatimosim E, Crous PW (2013) The *Colletotrichum orbiculare* species complex: Important pathogens of field crops and weeds. Fungal Diversity 61: 29–59. <https://doi.org/10.1007/s13225-013-0255-4>

Damm U, Sato T, Alizadeh A, Groenewald JZ, Crous PW (2019) The *Colletotrichum dracaenophilum*, *C. magnum* and *C. orchidearum* species complexes. Studies in Mycology 92: 1–46. <https://doi.org/10.1016/j.simyco.2018.04.001>

Diao Y-Z, Zhang C, Liu F, Wang W-Z, Liu L, Cai L, Liu X-L (2017) *Colletotrichum* species causing anthracnose disease of chili in China. Persoonia 38: 20–37. <https://doi.org/10.3767/003158517X692788>

Farr DF, Aime MC, Rossman AY, Palm ME (2006) Species of *Colletotrichum* on *Agavaceae*. Mycological Research 110: 1395–1408. <https://doi.org/10.1016/j.mycres.2006.09.001>

Fu M, Crous PW, Bai Q, Zhang PF, Xiang J, Guo YS, Zhao FF, Yang MM, Hong N, Xu WX, Wang GP (2019) *Colletotrichum* species associated with anthracnose of *Pyrus* spp. in China. Persoonia 42: 1–35. <https://doi.org/10.3767/persoonia.2019.42.01>

Gan P, Tsushima A, Hiroyama R, Narusaka M, Takano Y, Narusaka Y, Kawaradani M, Damm U, Shirasu K (2019) *Colletotrichum shisoi* sp. nov., an anthracnose pathogen of *Perilla frutescens* in Japan: molecular phylogenetic, morphological and genomic evidence. Scientific Reports 9: 13349. <https://doi.org/10.1038/s41598-019-50076-5>

Liu F, Cai L, Crous PW, Damm U (2013) Circumscription of the anthracnose pathogens *Colletotrichum lindemuthianum* and *C. nigrum*. Mycologia 105: 844–860. <https://doi.org/10.3852/12-315>

Liu F, Cai L, Crous PW, Damm U (2014) The *Colletotrichum gigasporum* species complex. Persoonia 33: 83–97. <https://doi.org/10.3767/003158514X684447>

Liu L-P, Wang Y, Qiu P-L, Zhang B, Zhang L, Wang N, Li Y, Gao J, Hsiang T (2020) *Colletotrichum neorubicola* sp. nov., a new leaf anthracnose pathogen of raspberry from northeast China. Mycological Progress 19: 947–955. <https://doi.org/10.1007/s11557-020-01614-3>

Marin-Felix Y, Groenewald JZ, Cai L, Chen Q, Marincowitz S, Barnes I, Bensch K, Braun U, Camporesi E, Damm U, de Beer ZW, Dissanayake A, Edwards J, Giraldo A, Hernández-Restrepo M, Hyde KD, Jayawardena RS, Lombard L, Luangsa-Ard J, McTaggart AR, Rossman AY, Sandoval-Denis M, Shen M, Shivas RG, Tan YP, van der Linde EJ, Wingfield MJ, Wood AR, Zhang JQ, Zhang Y, Crous PW (2017) Genera of phytopathogenic fungi: GOPHY 1. Studies in Mycology 86: 99–216. <https://doi.org/10.1016/j.simyco.2017.04.002>

New York State Museum (1909) Museum Bulletin No. 131. University of the State of New York, Albany, NY, 81 pp. <https://www.biodiversitylibrary.org/page/35126737>

O’Connell RJ, Thon MR, Hacquard S, Amyotte SG, Kleemann J, Torres MF, Damm U, Buiate EA, Epstein L, Alkan N, Altmüller J, Alvarado-Balderrama L, Bauser CA, Becker C, Birren BW, Chen Z, Choi J, Crouch JA, Duvick JP, Farman MA, Gan P, Heiman D, Henrissat B, Howard RJ, Kabbage M, Koch C, Kracher B, Kubo Y, Law AD, Lebrun M-H, Lee Y-H, Miyara I, Moore N, Neumann U, Nordström K, Panaccione DG, Panstruga R, Place M, Proctor RH, Prusky D, Rech G, Reinhardt R, Rollins JA, Rounsley S, Schardl CL, Schwartz DC, Shenoy N, Shirasu K, Sikhakolli UR, Stüber K, Sukno SA, Sweigard JA, Takano Y, Takahara H, Trail F, van der Does HC, Voll LM, Will I, Young S, Zeng Q, Zhang J, Zhou S, Dickman MB, Schulze-Lefert P, Ver Loren van Themaat E, Ma L-J, Vaillancourt LJ (2012) Lifestyle transitions in plant pathogenic *Colletotrichum* fungi deciphered by genome and transcriptome analyses. Nature Genetics 44: 1060–1065. <https://doi.org/10.1038/ng.2372>

Rakotoniriana EF, Scauflaire J, Rabemanantsoa C, Urveg-Ratsimamanga S, Corbisier A-M, Quetin-Leclercq J, Declerck S, Munaut F (2013) *Colletotrichum gigasporum* sp. nov., a new species of *Colletotrichum* producing long straight conidia. Mycological Progress 12: 403–412. <https://doi.org/10.1007/s11557-012-0847-5>

Sui X, Guo M, Wang Q, Wang H, Zhou H, Huang X, Yu Z, Hou C (2024) Taxonomy and phylogeny of *Colletotrichum* on important forest plants in China. Mycosphere 15: 4569–4743. <https://doi.org/10.5943/mycosphere/15/1/19>

Thao LD, Choi H, Choi Y, Mageswari A, Lee D, Kim D-H, Lee SH, Shin H-D, Damm U, Hong S-B (2024) The *Colletotrichum destructivum* species complex in Korea, description of *C. kummerowiae* sp. nov. and neotypification of *C. panacicola*. Mycological Progress 23: 43. <https://doi.org/10.1007/s11557-024-01982-0>

Wang C-B, Jiang N, Xue H, Piao C-G, Li Y (2022) *Colletotrichum chinense* sp. nov. from *Yucca gloriosa* and *C. quercicola* sp. nov. from *Quercus variabilis* in China. MycoKeys 93: 1–21. <https://doi.org/10.3897/mycokeys.93.89209>

Wikee S, Cai L, Pairin N, McKenzie EHC, Su Y-Y, Chukeatirote E, Thi HN, Bahkali AH, Moslem MA, Abdelsalam K, Hyde KD (2011) *Colletotrichum* species from jasmine (*Jasminum sambac*). Fungal Diversity 46: 171–182. <https://doi.org/10.1007/s13225-010-0049-x>

Xu H, Zhou R, Fu J, Yuan Y, Ge X, Damm U (2018) *Colletotrichum atractylodicola* sp. nov.: the anthracnose pathogen of *Atractylodes chinensis* in China. Mycological Progress 17: 393–402. <https://doi.org/10.1007/s11557-017-1359-0>

Yu Z, Jiang X, Zheng H, Zhang H, Qiao M (2022) Fourteen new species of foliar *Colletotrichum* associated with the invasive plant *Ageratinaadenophora* and surrounding crops. Journal of Fungi 8: 185. <https://doi.org/10.3390/jof8020185>

Zhang W, Fan X (2025) Diversity and distribution of *Colletotrichum* species causing anthracnose in China. Journal of Fungi 11: 781. <https://doi.org/10.3390/jof11110781>
